# Supplementary material for: Pharmacovigilance in Pediatric Patients with Epilepsy Using Antiepileptic Drugs
Source: Int J Environ Res Public Health. 2022 Apr 8;19(8):4509. doi: 10.3390/ijerph19084509 (PMC9028571; doi:10.3390/ijerph19084509)
Supplement: Supplementary file 1 [file ijerph-19-04509-s001.zip › ijerph-1651674-supplementary.pdf]

Questionnaire

Please, indicate an adverse drug reaction that occurs within the last 3 months (multiple choices possible).

a. Emotional liability
b. Fatigue
c. Psychomotor agitation
d. Agressivity
e. Anxiety
f. Headache
g. Hair loss
h. Skin reactions
i. Diplopia or blurred vision
j. Dyspepsia
k. Gingival hypertrophy
l. Tremor
m. Weight gain
n. Dizziness
o. Somnolence
p. Memory impairment
q. Sleep disturbance
r. Lack of concentration
s. Other…………………………………
………………………………………
…………………………………
